# Washington City Dental Society

**Published:** 1896-03

**Authors:** 


### Washington City Dental Society.

At the twenty-ninth annual banquet of the Washington City Dental Society, held December 19th, at the Cochran, Dr. A. W. Sweeney read the annual address and Dr. L. C. F. Hugo officiated as toastmaster, while the following responded to toasts: Dr. Wm. M. Hunt, Dr. H. B. Noble, Dr. H. C. Thompson, Dr. Wm. S. Donnelly, Dr. G. L. Hills, Dr. M. F. Finley, Dr. A. W. Sweeney, J. Roland Walton and C. H. Lyme. Officers for the ensuing year were elected as follows: Dr. William M. Hunt, President; Dr. D. E. Wiber, Vice-President; Dr. W. N. Cogan, Secretary; Dr. M. F. Finley, Treasurer; Dr. N. H. B. Noble, Librarian, and Dr. Garnett L. Hills, Essayist.

